# Antibiofilm peptides enhance the corrosion resistance of titanium in the presence of *Streptococcus mutans*

**DOI:** 10.3389/fbioe.2023.1339912

**Published:** 2024-01-10

**Authors:** Dan Wang, Yingying Yue, He Liu, Tian Zhang, Evan F. Haney, Robert E. W. Hancock, Jian Yu, Ya Shen

**Affiliations:** ^1^ Department of Stomatology, Tongji Hospital, Tongji Medical College, Huazhong University of Science and Technology, Wuhan, China; ^2^ Division of Endodontics, Department of Oral Biological and Medical Sciences, Faculty of Dentistry, University of British Columbia, Vancouver, BC, Canada; ^3^ Liaoning Institute of Science and Technology, Benxi, China; ^4^ School of Medicine, Vanderbilt University, Nashville, TN, United States; ^5^ Centre for Microbial Diseases and Immunity Research, Department of Microbiology and Immunology, University of British Columbia, Vancouver, BC, Canada; ^6^ State Key Laboratory of Oral and Maxillofacial Reconstruction and Regeneration, Key Laboratory of Oral Biomedicine Ministry of Education, Hubei Key Laboratory of Stomatology, School and Hospital of Stomatology, Wuhan University, Wuhan, China

**Keywords:** biofilm, corrosion, implant, peptide, titanium

## Abstract

Titanium alloys have gained popularity in implant dentistry for the restoration of missing teeth and related hard tissues because of their biocompatibility and enhanced strength. However, titanium corrosion and infection caused by microbial biofilms remains a significant clinical challenge leading to implant failure. This study aimed to evaluate the effectiveness of antibiofilm peptides 1018 and DJK-5 on the corrosion resistance of titanium in the presence of *Streptococcus mutans*. Commercially pure titanium disks were prepared and used to form biofilms. The disks were randomly assigned to different treatment groups (exposed to *S. mutans* supplied with sucrose) including a positive control with untreated biofilms, peptides 1018 or DJK-5 at concentrations of 5 μg/mL or 10 μg/mL, and a negative control with no *S. mutans*. Dynamic biofilm growth and pH variation of all disks were measured after one or two treatment periods of 48 h. After incubation, the dead bacterial proportion, surface morphology, and electrochemical behaviors of the disks were determined. The results showed that peptides 1018 and DJK-5 exhibited significantly higher dead bacterial proportions than the positive control group in a concentration dependent manner (*p* < 0.01), as well as far less defects in microstructure. DJK-5 at 10 μg/mL killed 84.82% of biofilms and inhibited biofilm growth, preventing acidification due to *S. mutans* and maintaining a neutral pH. Potential polarization and electrochemical impedance spectroscopy data revealed that both peptides significantly reduced the corrosion and passive currents on titanium compared to titanium surfaces with untreated biofilms, and increased the resistance of the passive film (*p* < 0.05), with 10 μg/mL of DJK-5 achieving the greatest effect. These findings demonstrated that antibiofilm peptides are effective in promoting corrosion resistance of titanium against *S. mutans*, suggesting a promising strategy to enhance the stability of dental implants by endowing them with antibiofilm and anticorrosion properties.

## 1 Introduction

On account of its excellent biocompatibility, mechanical strength, and low modulus of elasticity, titanium has been extensively applied in implant dentistry for the restoration of missing teeth and hard tissues in recent decades (López-Píriz et al., 2019). However, oral bacteria from dental tissue inflammatory sites can adhere to any surface of materials placed in the oral cavity, including titanium. As a result, bacterial biofilm formation on titanium remains a significant clinical challenge, leading to peri-implantitis and the failure of dental implantation (Daubert and Weinstein, 2019; Dhaliwal et al., 2021; Siddiqui et al., 2022). When exposed to fermentable carbohydrates, acid-producing bacteria within biofilms, such as *Streptococcus mutans*, can generate a low pH microenvironment via bacterial metabolism, and electrochemical circuits via a potential difference (Pitts et al., 2017; Yu et al., 2022). This facilitates the deterioration of protective passive oxide film of titanium via a bio-corrosion process, and the demineralization of dental hard tissues (Prestat and Thierry, 2021; Weller et al., 2022). Titanium corrosion is likely to result in the release of titanium ions, causing an inflammatory reaction in the surrounding tissues, which is regarded as another critical risk factor for implant failure (Curtin and Wang, 2017; Prestat and Thierry, 2021; Alhamad et al., 2023). Accordingly, effective strategies are necessary to improve the antibacterial ability and corrosion resistance of titanium, thereby prolonging its stability and service life.

Controlling the inflammation process is the main purpose and principle for the treatment of titanium corrosion and peri-implant diseases. To address this issue, either surgical or nonsurgical approaches are generally employed in clinical practice (Hasan et al., 2022). Noninvasive strategies provide nonsurgical therapy with enormous potential for the treatment of implant-associated infections. However, owing to implant design, it is unlikely to completely remove the biofilms by nonsurgical mechanical therapy alone (Renvert et al., 2008). The adjunctive application of antimicrobials, including chemical agents and antibiotics, is therefore considered a promising strategy to alleviate microbial burden (Patil et al., 2022; López-Valverde et al., 2023). The past few years have witnessed a variety of antimicrobial agents for use in implant disinfection (Malheiros et al., 2023). Nevertheless, due to heterogeneous microbial community organization, as well as existing extracellular polymeric substances and differentiated gene expression, dental biofilms are recalcitrant to most antibiotics and difficult to treat (Kim et al., 2014; Wang et al., 2015). In this regard, microbial overgrowth is likely to increase the corrosion risk of titanium and affect the homeostasis of normal oral flora.

Recent studies have suggested that cationic antimicrobial peptides (host defense peptides) are effective as potential alternatives in treating biofilm-associated infections (Gera et al., 2022; Shao et al., 2023). By specifically targeting microbial biofilms with these peptides, pre-existing biofilms can be dissolved and biofilm formation can be prevented (de la Fuente-Núñez et al., 2012). Their unique additional ability to modulate the immune system contributes significantly to microbial killing and reducing local inflammation (Mansour et al., 2014). Recently, broad-spectrum antibiofilm peptides 1018 and DJK-5 (Supplementary Figure S1) have been shown to act by promoting guanosine tetraphosphate (ppGpp) degradation, which is crucial for biofilm growth (de la Fuente-Núñez et al., 2014; de la Fuente-Núñez et al., 2015). While peptide 1018 comprises natural L-amino acids, peptide DJK-5 contains D-amino acids that alter the peptide stereochemistry and confers proteolytic stability (de la Fuente-Núñez et al., 2015). Both oral single-species and multispecies biofilms formed on dental substrates have been shown to be killed by these two peptides in our previous investigations (Wang et al., 2018; Huang et al., 2019). These advantages imply that antibiofilm peptides might be promising for application in preventing titanium corrosion caused by microbial biofilms. No published data has reported the effects of peptides 1018 and DJK-5 on the corrosion behaviors of titanium against biofilms at present.

The objective of this study was to evaluate the effectiveness of antibiofilm peptides 1018 and DJK-5 on the corrosion resistance of titanium exposed to *S. mutans*. The null hypotheses were that (i) peptides 1018 and DJK-5 cannot kill *S. mutans* biofilm on titanium surfaces in one or two 48-h periods; (ii) peptides 1018 and DJK-5 cannot enhance the corrosion resistance of titanium in one or two 48-h periods; and (iii) there is no difference in the efficacy of peptides 1018 and DJK-5 on biofilm killing and corrosion resistance.

## 2 Materials and methods

### 2.1 Preparation of titanium disks

Commercial pure titanium (cp-Ti, grade 2, Baoji Titanium Industry Co., Ltd., Baoji, China) was used to prepare titanium disks with a diameter of 12 mm and a thickness of 1.5 mm. All disks were uniformly polished with silicon carbide sandpapers ranging from 400 to 1,200 grit in sequence and then cleaned by ultrasonic bathing in deionized water, acetone, 75% ethanol, and deionized water for 10 min, respectively. After drying overnight at 60°C, these disks were sterilized by UV light for 6 h on each side and placed in sterile 24-well plates.

### 2.2 Energy dispersive X-ray spectroscopy (EDS) analysis of titanium disks

The elemental analysis of titanium disks was detected by EDS (Helios Nanolab 650, FEI, Eindhoven, Netherlands) to inspect their purity. Three titanium disks were randomly selected, and three random areas on each disk were recorded and measured by EDS at a voltage of 10 kV.

### 2.3 Synthesis of the peptides

As described in previous studies (Yang et al., 2019; Hu et al., 2023), peptides DJK-5 and 1018 were synthesized in the method of solid-phase 9-fluorenylmethoxy carbonyl (Fmoc) chemistry by CPC Scientific (Sunnyvale, United States). The synthesized peptides were identified by amino acid analysis, and reverse-phase high-performance liquid chromatography was utilized to achieve at least 95% purity. One hundred μg/mL stock solutions of both peptides were then prepared by dissolving lyophilized powders in sterile deionized water and keeping them sterile over time.

### 2.4 Bacterial strain and culture condition

*S. mutans* (ATCC 25175) was grown anaerobically on plates with 32 g/L of brain-heart infusion (BHI) agar (Becton-Dickinson, Sparks, United States) at 37°C and subcultured on a new agar plate for 48–72 h. After that, the bacteria were picked up and transferred into BHI broth (Becton-Dickinson, Sparks, United States) for 24 h of incubation at 37°C. Amplified bacteria were harvested by centrifugation (5,000 rpm) at 4°C for 5 min and washed twice with PBS. The collected *S. mutans* were suspended in BHI to achieve an optical density (OD) at 0.05 as determined by a microplate reader (ELx808, BioTek, Winooski, United States) at the wavelength of 405 nm.

### 2.5 Biofilm formation on titanium disks

Titanium disks were placed in 24-well plates (one disk in each well) and randomly distributed into different groups in accordance with the following treatments: 1) the positive control (untreated biofilm) group: bacterial suspension (200 μL) and BHI (1,800 μL) containing sucrose (222.22 g/L) were added to each well; 2) 5 μg/mL of peptide treatment groups (DJK-5 or 1018): peptide stock solution (100 μL), bacterial suspension (200 μL), and BHI (1,700 μL) containing sucrose (235.29 g/L) were added to each well; 3) 10 μg/mL of peptide treatment groups (DJK-5 or 1018): peptide stock solution (200 μL), bacterial suspension (200 μL), and BHI (1,600 μL) containing sucrose (250 g/L) were added to each well; and 4) the negative control group: each well only added with BHI (2,000 μL) containing sucrose (200 g/L). Each group of bacteria received the same amount of sucrose (200 g/L as the final concentration (Souza et al., 2010; Souza et al., 2013).

Two different incubation times were tested separately for each experiment condition. In the 48 h group, titanium disks were removed from each well after 48 h and rinsed three times with sterile deionized water, followed by a 5-min ultrasonic bath in 100% ethanol and air drying. In the two 48-h treatments group, the consumed solution was removed after incubation for the first 48 h, and the corresponding fresh peptide solution and BHI medium were supplied for another 48 h. Following that, titanium disks were rinsed and dried as previously stated.

### 2.6 Dynamic monitoring of biofilm development and pH value

To monitor the dynamic growth of *S. mutans* biofilms on titanium disks treated with or without peptides DJK-5 and 1018, biofilms were detached at designated time intervals (0, 2, 4, 6, 8, 10, 12, 18, 24, 30, 36, 48, 72, and 96 h) and dispersed by pipetting to obtain an even bacterial suspension. Subsequently, 150 μL of the suspension was aspirated into 96-well plates and read by the microplate reader at OD_405nm_. Three parallel wells were replicated at each time interval for each group. Then, the pH of the remaining suspension in each well was measured by a pH Meter (Accumet Basic AB 15 Plus, Thermo Fisher Scientific, Waltham, United States). For each group, three parallel wells were tested and recorded.

### 2.7 Determination of dead bacterial proportion

After incubation for one or two 48-h periods, titanium disks coated with biofilms were gently washed with PBS and stained with a fresh mixture of SYTO-9 and propidium iodide (BacLight live/dead bacterial viability kit, Molecular Probes, Eugene, United States) based on the manufacturer’s instruction. And observed by confocal laser scanning microscopy (CLSM, FV10i-LIV, Olympus, Canada) (Yu et al., 2021; Liu et al., 2022). The wavelength of excitation/emission light detected for SYTO-9 and propidium iodide was set 480/500 nm and 490/635 nm, respectively. For each group, three disks were selected, and five areas for each disk were scanned from the bottom to the top of the biofilm with a 2 μm step. An Imaris 7.2 software (Bitplane, Switzerland) was employed to convert the two-dimension images into three-dimensional volume stacks. The obtained green and red fluorescence intensity was analyzed as the total volume of live and dead bacteria, and the dead bacteria proportion was calculated using red fluorescence in relation to total fluorescence (green + red) (Guo et al., 2021).

### 2.8 Scanning electron microscopy (SEM) of corrosion characters

The characteristics of corrosive titanium surfaces exposed to *S. mutans* (treated with or without peptides DJK-5 and 1018) were inspected through SEM (SU3500, Hitachi, Toronto, Canada). After one or two 48-h periods of incubation, titanium disks were taken out from the wells and washed three times using sterile deionized water to detach the biofilms before being ultrasonically cleaned for 5 min in sterile deionized water and 100% ethanol, respectively. After drying in the air, randomly selected areas from SEM images (at ×3,000) were captured at 3 kV for each disk.

### 2.9 Electrochemical tests in artificial saliva

**FIGURE 1:**
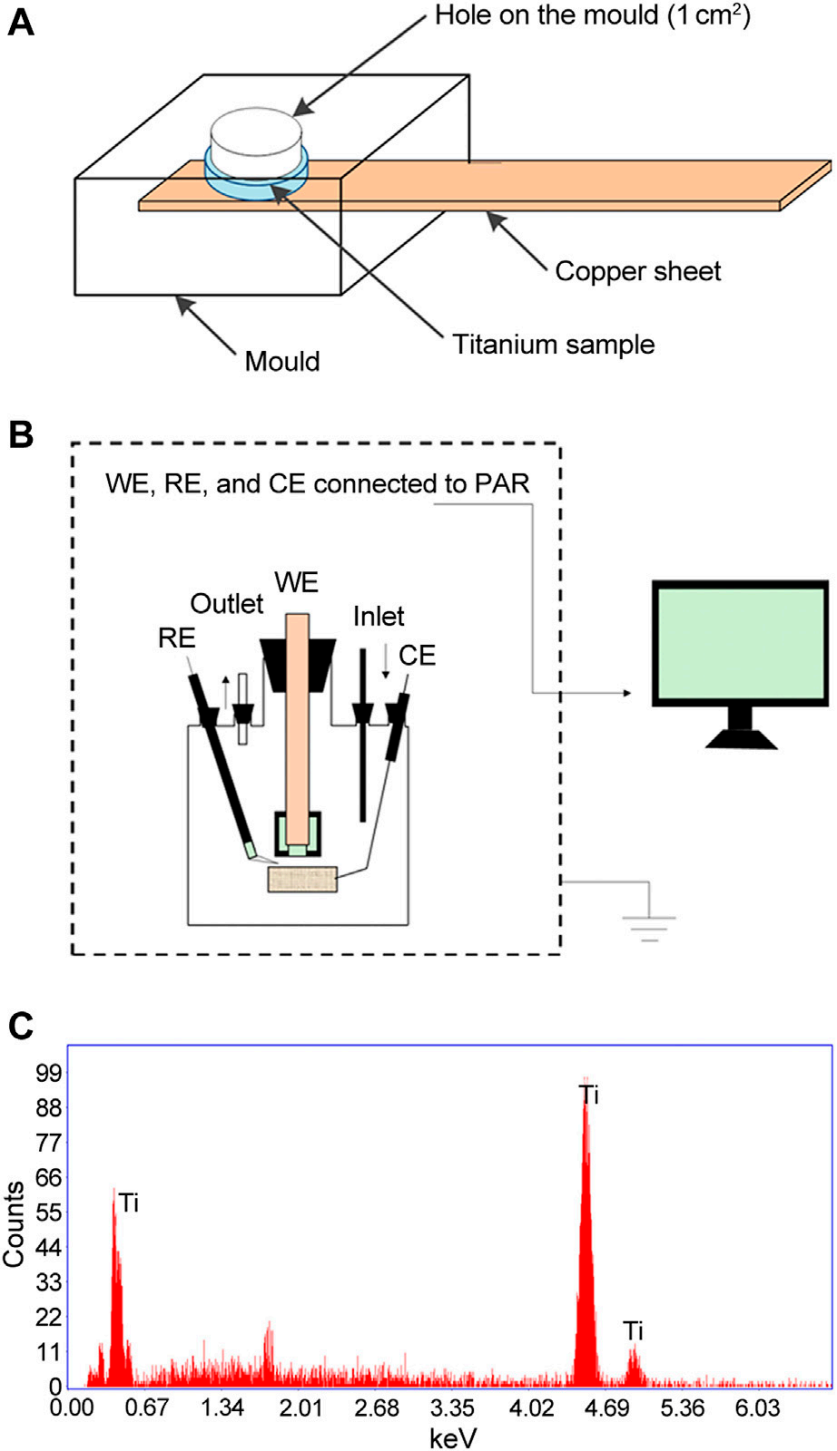
Schematic diagrams of **(A)** The mould and **(B)** The electrochemical setup used in the present study. **(C)** EDS analysis of titanium disks used prior to corrosion exposure. CE, counter electrode; RE, reference electrode; WE, working electrode.

The electrochemical experiments were carried out to investigate the effects of DJK-5 and 1018 on the corrosion resistance of titanium after exposure to *S. mutans*. A Princeton Applied Research (PAR) VersaSTAT 4 potentiostat (AMETEK, Berwyn, United States) was employed in this study. The electrochemical setup consisted of a conventional three-electrode cell with a platinum net as a counter electrode (CE) and an Ag/AgCl [(KCl) = 4 M] electrode as a reference electrode (RE) introduced into the cell through a Luggin probe. All potential values are used according to the Ag/AgCl reference electrode (0.197 V vs. SHE). The working electrode (WE) was set in a special mould with an exposed area of 1 cm^2^ as the working area. The schematic diagram of the mould and the electrochemical setup are presented in Figures 1A, B. The biofilm-coated disks were connected to the PAR via a copper sheet. The solutions were purged with nitrogen for at least 30 min before introducing the working electrode, and the purging lasted throughout the experiment. Each test was repeated at least three times to ensure reproducibility.

The electrochemical tests were performed in a modified Fusayama’s artificial saliva solution (0.4 g/L NaCl, 0.4 g/L KCl, 0.795 g/L CaCl_2_·2H_2_O, 0.005 g/L Na_2_S·9H_2_O, 0.69 g/L NaH_2_PO4·H_2_O, 1.0 g/L urea, pH = 6.0) at 37°C ± 0.5°C as previously reported (Souza et al., 2013; Fukushima et al., 2018). The potential dynamic polarization (PDP) curves and electrochemical impedance spectroscopy (EIS) tests were determined to evaluate the corrosion resistance of titanium in the Fusayama’s artificial saliva after different treatments (Zhang et al., 2007). Prior to the tests, the open circuit potential (OCP) measurements were carried out for at least 1 h until it reached a quasi-steady state value (deviation in OCP from its 5 min value within 2%). PDP measurements were conducted from −0.5 V vs. OCP to 1.5 V vs. OCP at a scan rate of 1 mV/s. The electrochemical parameters, including the corrosion potential (*E*_corr_), corrosion current density (*I*_corr_), pitting potential (*E*_pit_), and passive current density (*I*_passive_), were determined. EIS measurements were conducted at OCP with a sinusoidal potential wave as a disturbing signal. The amplitude of sinusoidal potential was 10 mV, and the scan frequency ranged from 0.01 to 105 Hz. The EIS results were analyzed with ZSimpWin software (AMETEK, Berwyn, United States). All experiments were performed with at least three independent tests.

### 2.10 Statistical analysis

All data obtained were presented as means ± standard deviations for statistical analysis. One-way analysis of variance (ANOVA) was performed by SPSS 22.0 software (IBM, Armonk, United States), and *post hoc* Fisher’s LSD multiple comparison tests were then examined. The significance level was set at *p* < 0.05.

## 3 Results

### 3.1 EDS analysis

The elemental content of titanium disks used in this study was examined by EDS. As shown in Figure 1C, almost all of the typical titanium element peaks were detected. The results of the EDS analysis revealed that titanium had a purity of more than 99.9%, as measured by the atomic percentage of the Ti element.

### 3.2 Dynamic determination of biofilm development and pH value

**FIGURE 2:**
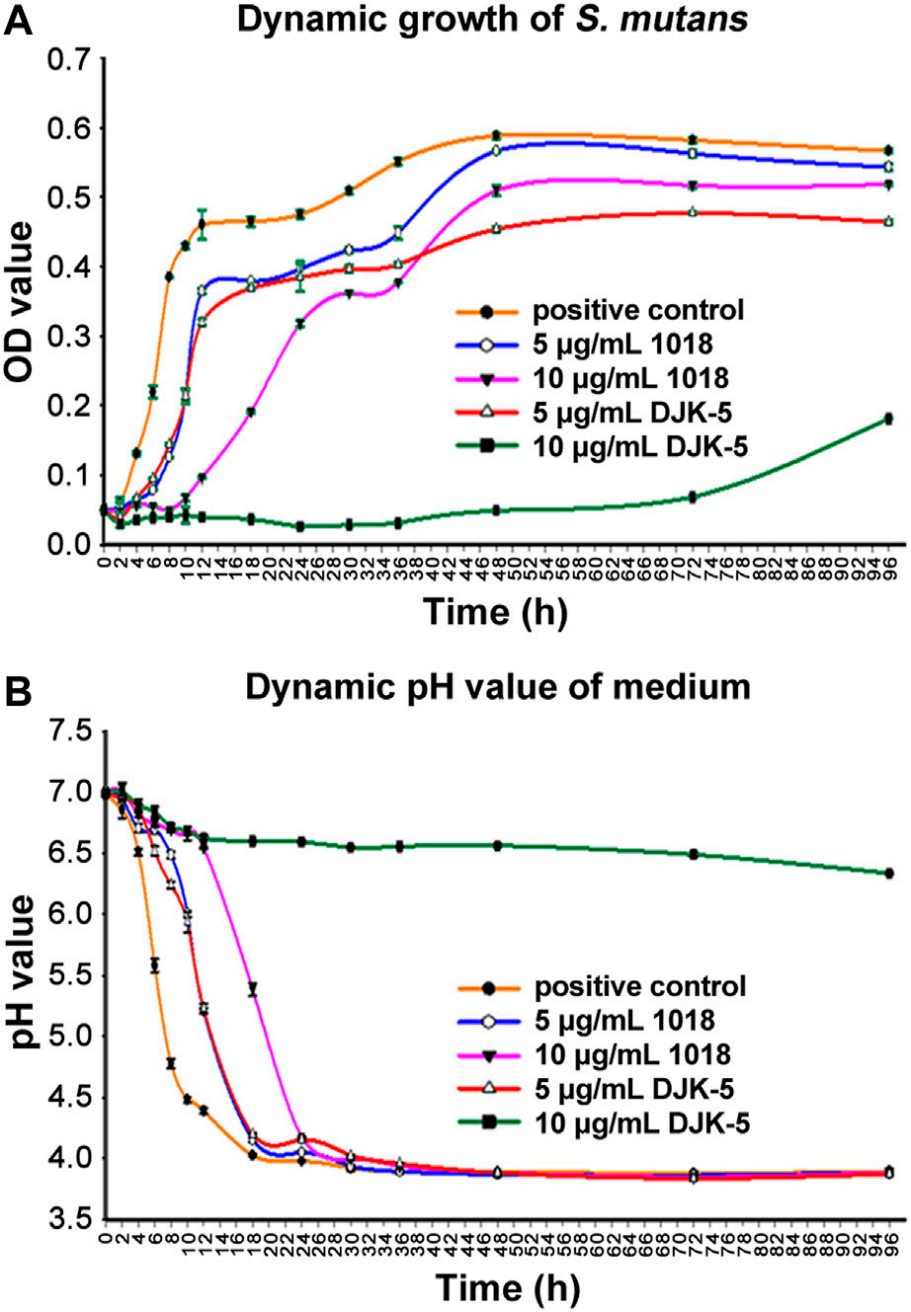
The dynamic determination of *S. mutans* in BHI medium supplemented with sucrose (200 g/L) from 0 to 96 h for each group: **(A)** The dynamic growth of *S. mutans* biofilms on titanium disks as measured by optical density at 405 nm; and **(B)** The dynamic variation of the pH of the culture medium. Data are shown as means ± standard deviations (no statistical analysis was carried out).

The dynamic growth of *S. mutans* biofilm on titanium disks in each group was plotted in Figure 2A. In the first 2 h, there was no discernible increase in bacteria. Then, *S. mutans* proliferation in the positive control group accelerated and reached ∼9-fold the initial level after 12 h, followed by a gradual increase up to 48 h and then levels plateaued. *S. mutans* exposed to 5 μg/mL of peptide 1018 or DJK-5 showed similar but delayed kinetics with a longer lag phase, beginning to thrive after 4 h and lower growth by 6–7 fold in the first 8 h. Bacterial growth was stabilized between 12 and 48 h and reached a plateau within 96 h. Overall, there were more bacteria in the 1018 group (5 μg/mL) than in the DJK-5 group (5 μg/mL) at each time interval. The kinetics of growth was much more dramatically affected at 10 μg/mL of peptide. Treatment with 10 μg/mL of 1018 led to much slower proliferation of *S. mutans* in the group between 12 and 48 h, but its eventual optical density after 48 h value was higher than in the 5 μg/mL of DJK-5 group. The biofilm development of *S. mutans* exposed to 10 μg/mL of DJK-5 was substantially and effectively inhibited during the first 72-h, but increased slightly by 96 h.

*S. mutans* is known acidify the medium. The pH of the medium in the untreated *S. mutans* control decreased to around 4 within 18 h, due to biofilm growth (Figure 2B). The decrease in pH values in each group at the designated time intervals corresponded to the increase in OD values. Specifically, in the positive control group, pH values dropped rapidly from 7.0 at 0 h to 4.48 within 10 h, then to 4.02 by 18 h, and then remained stable. The pH values of the medium with 5 μg/mL of 1018 and DJK-5 added revealed the same declining trend. They maintained a slower decline in pH than the positive control group but still reached a pH of around 4 by 18 h. Ten μg/mL of 1018 delayed the pH drop for the first 12 h, but it still went down to 4.17 at 24 h. Remarkably, 10 μg/mL of DJK-5 was able to maintain the pH around neutrality with only a 0.5 unit decrease during the 96-h incubation period.

### 3.3 Biofilm killing

**FIGURE 3:**
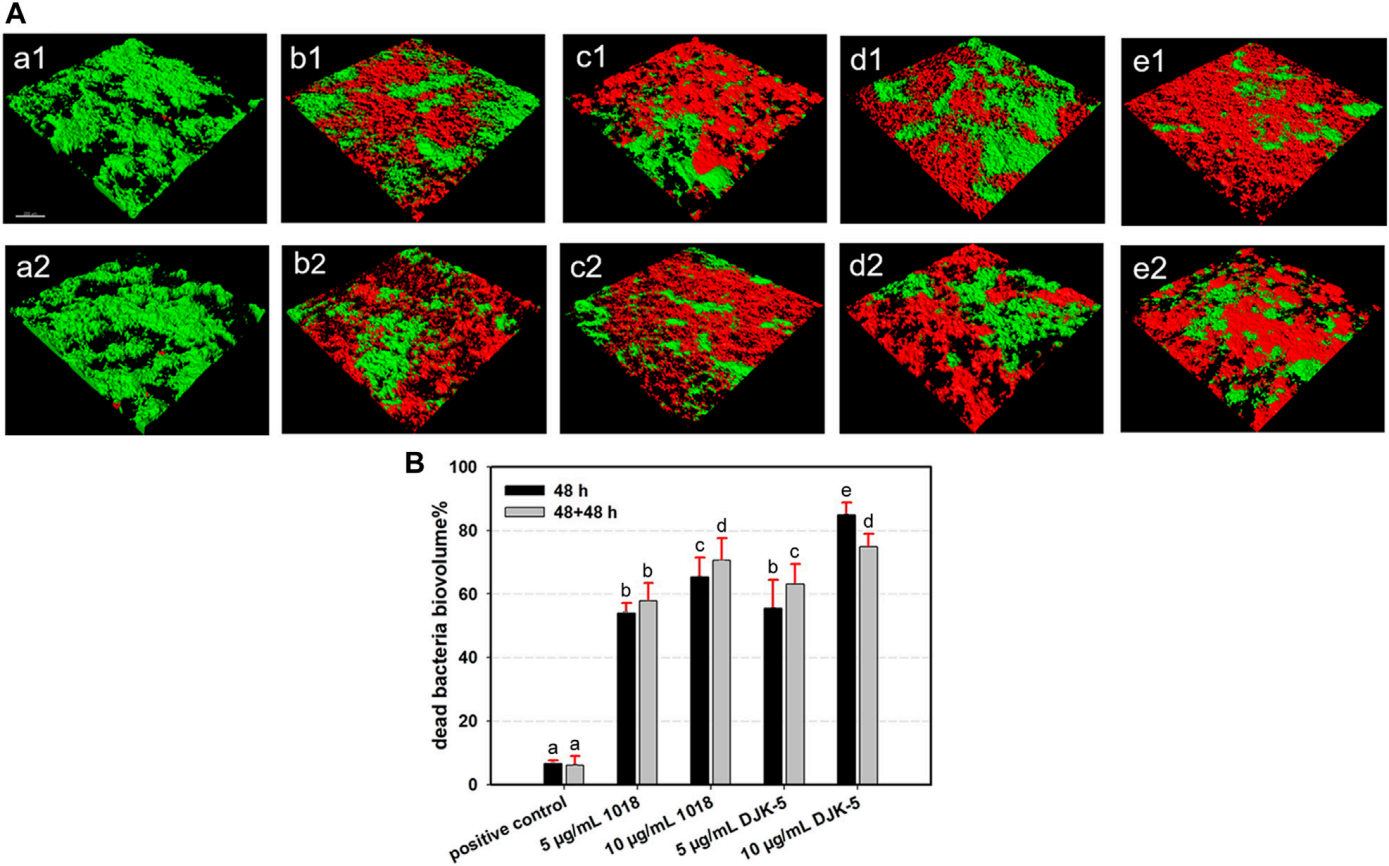
**(A)** Representative CLSM images (green, live bacteria; red, dead bacteria) and **(B)** Dead bacterial biovolume percentages of *S. mutans* biofilms for each group after 48 and 48 + 48 h of incubation. Images of (a1 and a2) positive control, (b1 and b2) 5 μg/mL 1018, (c1 and c2) 10 μg/mL 1018, (d1 and d2) 5 μg/mL DJK-5, and (e1 and e2) 10 μg/mL DJK-5 groups. Images of (a1–e1) 48 h and (a2–e2) 48 + 48 h. Data are shown as means ± standard deviations [groups with the same lowercase letters are not statistically significant (*p >* 0.05)].

The dead bacterial proportions and live/dead bacteria CLSM images of *S. mutans* biofilms on titanium surfaces after incubation with or without peptides for one or two 48-h periods are shown in Figures 3A, B. Both peptides 1018 and DJK-5 (at 5 and 10 μg/mL) killed the majority of bacteria over 48 h (from 53.95% to 84.82%) with significant differences compared to the positive control group (*p* < 0.01), and the most bacterial killing was found in the 10 μg/mL of DJK-5 group. Peptide DJK-5 was more effective in killing *S. mutans* than peptide 1018 at the same concentration, and higher concentrations (10 μg/mL) of peptide led to a higher proportion of dead bacteria than lower concentrations (5 μg/mL) did (*p* < 0.05). Except for DJK-5 at 10 μg/mL, more *S. mutans* were killed in the peptide-treated groups after two 48-h treatments than a single 48-h treatment. Ten μg/mL of DJK-5 (48 + 48 h) induced approximately 73.88% of bacterial cell death and had a higher killing proportion than the 5 μg/mL of DJK-5 and the positive control groups (*p* < 0.05).

### 3.4 SEM of corrosive titanium surfaces

**FIGURE 4:**
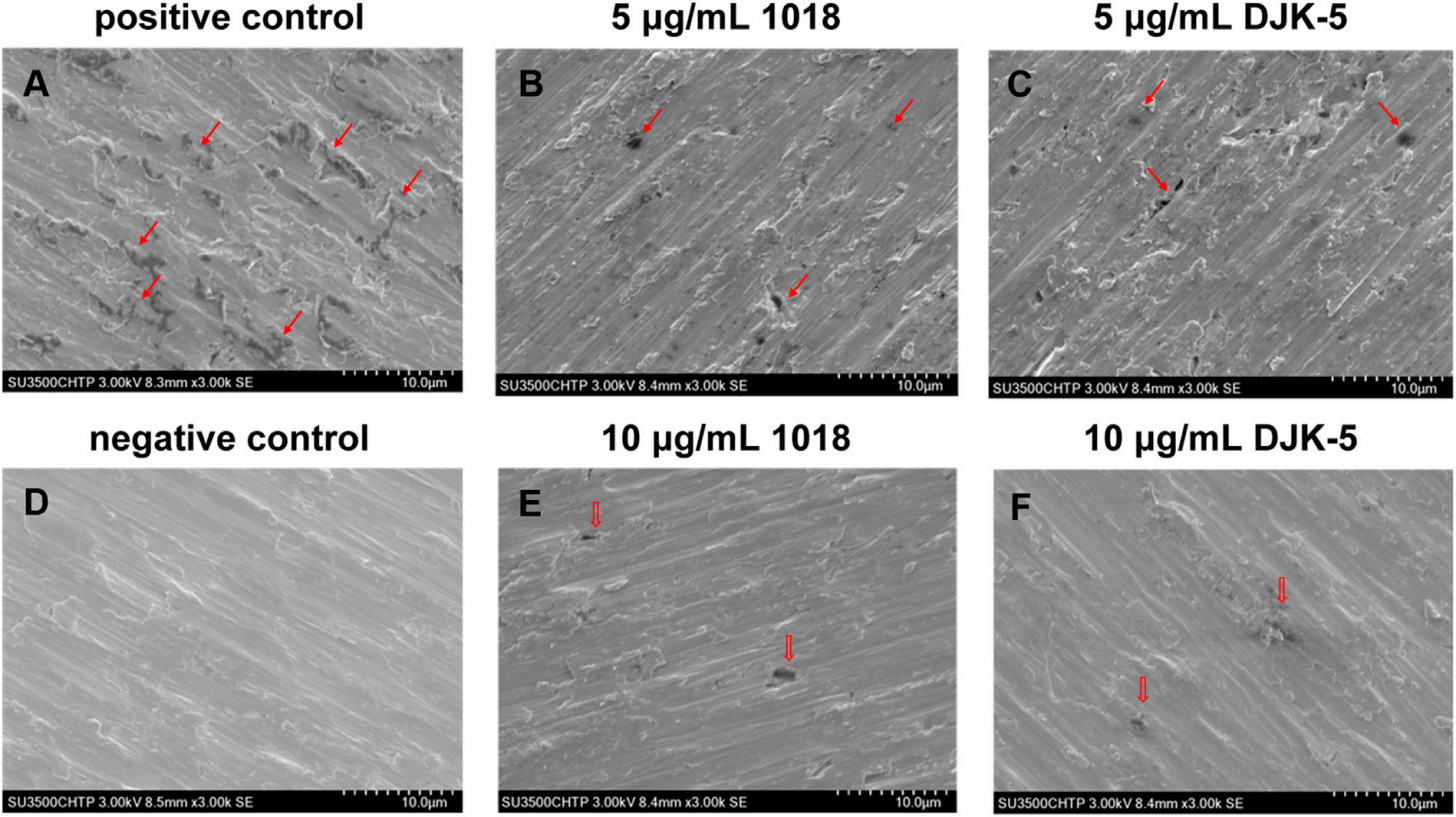
SEM images **(A–F)** of corrosion of titanium surfaces exposed to *S. mutans* in BHI medium supplemented with sucrose (200 g/L) for 48 + 48 h from each group (after biofilm removal). Red arrows indicate the corroded areas.

The SEM surface microstructures of titanium disks (after biofilm removal) from each group incubated with *S. mutans* for two 48-h treatments are shown in Figure 4. In the negative control group (no bacteria), the disks retained their smooth and even surface morphology, with the presence of longitudinal grooves as a consequence of the polishing process. In the positive control group, the titanium surface colonized by *S. mutans* from the beginning of immersion was rich in irregular micro-pits of various sizes and shapes along the longitudinal grooves, as indicated by red arrows in SEM images. In the case of titanium disks exposed to bacteria and 5 μg/mL of peptides 1018 and DJK-5, corrosion pits on their surfaces appeared to decrease. Ten μg/mL of peptide DJK-5 had the greatest ability to prevent material degradation, followed by 10 μg/mL of peptide 1018, with only a few visible randomly-scattered micro-pits.

### 3.5 Electrochemical results of PDP curves

**FIGURE 5:**
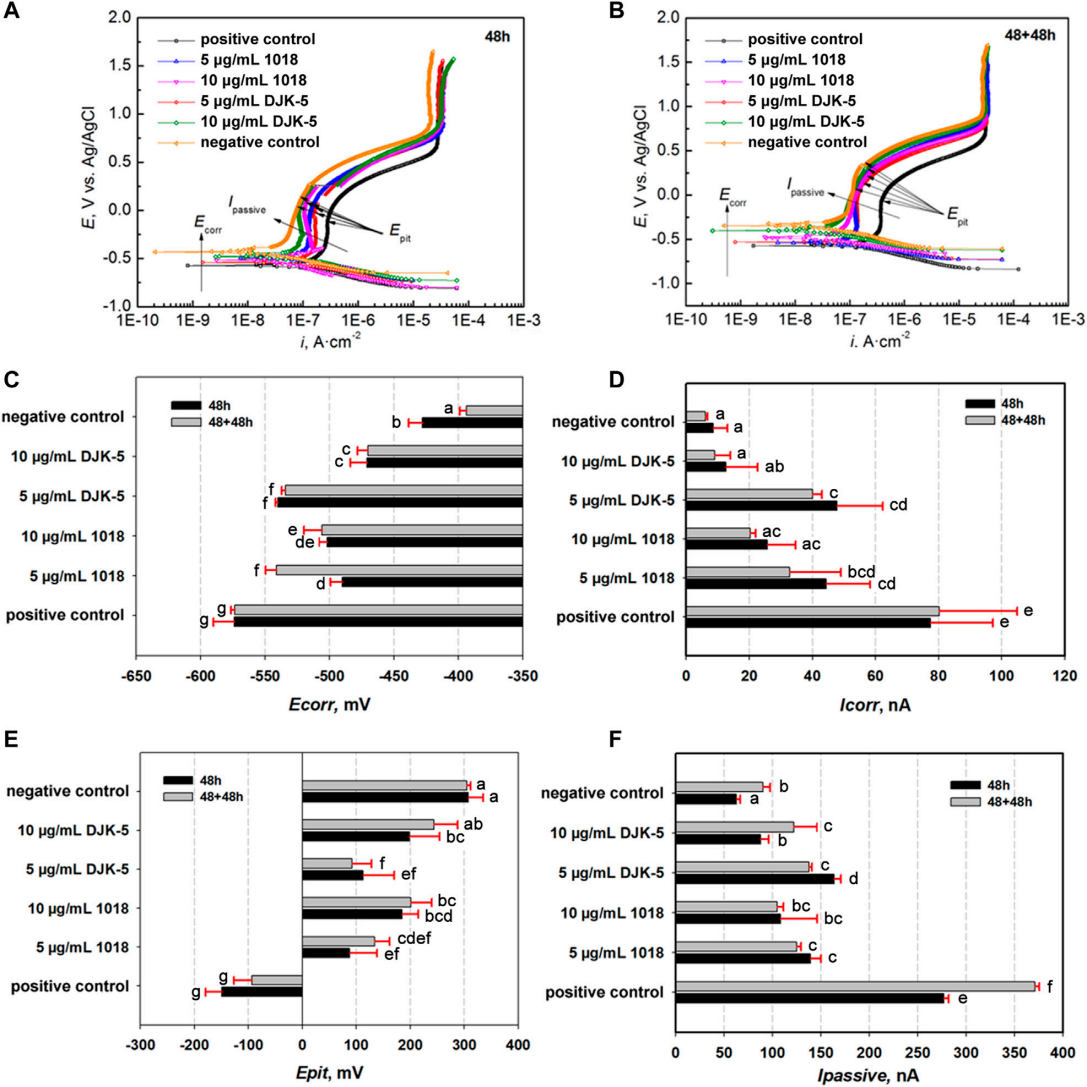
Potential dynamic polarization (PDP) curves of titanium disks after exposure to *S. mutans* with or without peptides for **(A)** 48 h and **(B)** 48 + 48 h, as well as **(C–F)** relative electrochemical parameters (*E*_corr_, *I*_corr_, *E*_pit_, and *I*_passive_). Data are shown as means ± standard deviations [groups with the same lowercase letters are not statistically significant (*p >* 0.05)].

To assess electrochemical corrosion resistance, PDP curves of titanium were examined in artificial saliva at 37°C after incubation with bacteria and peptides. As shown in Figures 5A, B, titanium disks demonstrated moderate corrosion resistance in the test environment because a typical passive plateau of 10^−7^ A·cm^−2^ was observed, followed by the *I*_passive_ ranging from 10^−6^ to 10^−7^ A·cm^−2^. This phenomenon reflected that a relatively protective passive film of titanium dioxide remained on the disks. The *E*_corr_ and *I*_corr_ in the active region were measured using the Tafel extension method. The passive film was broken down as the applied potential was increased above 10^−6^ A·cm^−2^, leading to a sharp increase in the current density. The specific electrochemical data of PDP are depicted in Figures 5C–F. The *E*_corr_ of the positive control group was lower than that of the other groups (*p* < 0.05), whereas the *I*_corr_ of the positive control group was higher than that of the other groups (*p* < 0.05). With the addition of peptides DJK-5 and 1018, the *E*_corr_ shifted to a less negative value while the *E*_pit_ showed significantly higher values than the positive control group (*p* < 0.05), and the *I*_corr_ and *I*_passive_ were significantly lower (*p* < 0.05).

After 48 h, the *I*_corr_ and *I*_passive_ had increased in the following order: negative control <10 μg/mL DJK-5 < 10 μg/mL 1018 < 5 μg/mL 1018 < 5 μg/mL DJK-5 < positive control. The OCP and *E*_pit_ decreased in the following order: negative control >10 μg/mL DJK-5 > 10 μg/mL 1018 > 5 μg/mL DJK-5 > 5 μg/mL 1018 > positive control. Treatment with 10 μg/mL of peptides 1018 and DJK-5 showed improved corrosion resistance of titanium when compared to 5 μg/mL of these peptides, with 10 μg/mL of DJK-5 achieving the greatest effect. A similar trend was observed for titanium disks after two treatments for 48 h of immersion, and the *I*_corr_ in each group after two 48-h treatments was lower than those after one treatment, except in the case of the positive control.

### 3.6 EIS measurements

**FIGURE 6:**
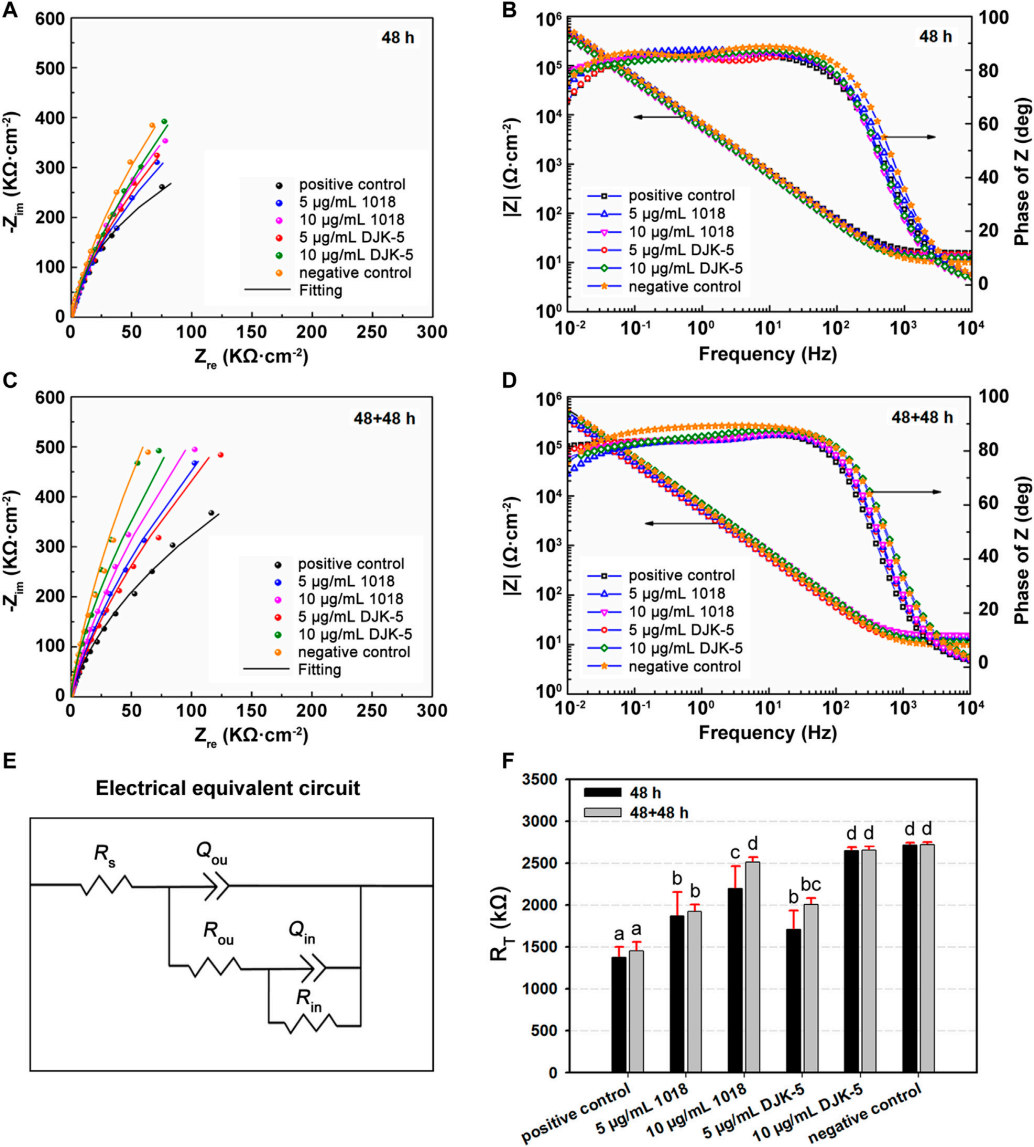
**(A,C)** Nyqusit and **(B,D)** Bode plots from EIS measurements for titanium disks in each group after incubation with *S. mutans* for **(A,B)** 48 h and **(C,D)** 48 + 48 h. **(E)** Electrical equivalent circuit used for fitting the impedance data. **(F)** *R*_T_ values of titanium disks for each group. Data are shown as means ± standard deviations [groups with the same lowercase letters are not statistically significant (*p >* 0.05)].

The EIS tests were performed to characterize the stability of the passive film on titanium after different treatments, with the Nyquist and Bode plots for each group in artificial saliva at 37°C are shown in Figures 6A–D. The Nyquist plots (Figures 6A, C) revealed a capacitive loop, whereas the Bode plots (Figures 6B, D) revealed only a one-time constant, suggesting that the corrosion process of titanium was controlled by charge transfer. The impedance values were in the order of 10^6^ Ω·cm^−2^, indicating that the titanium was covered by a protective passive film. The most commonly used electrical equivalent circuit (EEC) of titanium’s passive film is *R*_s_ {*Q*_ou_ [*R*_ou_ (*R*_in_ *Q*_in_)]}. *R*_s_ denotes the solution resistance, *Q*_ou_ and *Q*_in_ refer to the constant phase element (*Q*) of the outer porous film and inner barrier film, respectively, and *R*_ou_ and *R*_in_ represent the outer and inner film resistance. The resistance of the passive film (*R*_T_) equals *R*_ou_ + *R*_in_. The corresponding fitting data are listed in Table 1, with chi-square (χ^2^) values in the order of 10^−4^. The fitting curves, fitting results, and experimental data showed great agreement. Thus, the *R*_s_ {*Q*_ou_ [*R*_ou_ (*R*_in_ *Q*_in_)]} of EEC was appropriate to describe the passive film in this study.

**TABLE 1:** Means and (standard deviations) of electrochemical parameters determined by electrical equivalent circuit models for titanium disks from each group.

Time	Groups	*R*_s_ (Ω·cm^2^)	*Q*_ou_ × 10^5^ (s^−n^·Ω^−1^·cm^−2^)	*n*_1_	*R*_ou_ (kΩ·cm^2^)	*Q*_in_ × 10^5^ (s^−n^·Ω^−1^·cm^−2^)	*n*_2_	*R*_in_ (kΩ·cm^2^)	*R*_T_ (kΩ·cm^2^)
48 h	Positive control	12.15 ± 3.85	2.73 ± 0.56	1.00 ± 0.00	36.38 ± 20.92	0.72 ± 0.13	0.84 ± 0.13	1,339.67 ± 141.29	1,376.05 ± 122.71
	5 μg/mL 1018	12.04 ± 0.88	2.55 ± 0.34	1.00 ± 0.00	24.14 ± 14.53	2.42 ± 2.77	0.89 ± 0.07	1,847.00 ± 281.09	1,871.14 ± 286.18
	10 μg/mL 1018	10.10 ± 0.73	2.57 ± 0.34	1.00 ± 0.00	34.51 ± 31.23	0.69 ± 0.26	0.88 ± 0.07	2,163.67 ± 242.44	2,198.18 ± 266.59
	5 μg/mL DJK-5	13.05 ± 3.85	2.55 ± 0.67	1.00 ± 0.00	19.97 ± 14.02	0.82 ± 0.18	0.84 ± 0.03	1,689.67 ± 223.25	1,709.64 ± 227.40
	10 μg/mL DJK-5	11.51 ± 0.81	2.39 ± 0.27	0.99 ± 0.01	20.51 ± 14.96	0.84 ± 0.30	0.86 ± 0.11	2,627.00 ± 37.64	2,647.51 ± 39.83
	Negative control	15.27 ± 1.92	3.28 ± 0.75	1.00 ± 0.00	17.26 ± 12.87	1.15 ± 0.59	0.80 ± 0.03	2,698.33 ± 22.55	2,715.59 ± 30.34
48 + 48 h	Positive control	11.72 ± 2.95	2.30 ± 0.08	1.00 ± 0.00	18.24 ± 10.10	0.73 ± 0.14	0.84 ± 0.03	1,436.67 ± 106.93	1,454.91 ± 103.58
	5 μg/mL 1018	8.77 ± 2.17	2.83 ± 0.65	1.00 ± 0.00	31.66 ± 7.22	0.84 ± 0.25	0.83 ± 0.04	1,893.33 ± 77.67	1,924.99 ± 81.08
	10 μg/mL 1018	10.13 ± 3.01	2.39 ± 0.44	0.97 ± 0.00	22.15 ± 22.85	1.02 ± 0.50	0.85 ± 0.02	2,486.67 ± 80.83	2,508.82 ± 60.88
	5 μg/mL DJK-5	11.62 ± 3.45	2.67 ± 0.54	1.00 ± 0.00	21.15 ± 4.44	0.85 ± 0.19	0.83 ± 0.05	1,983.33 ± 76.38	2,004.48 ± 76.40
	10 μg/mL DJK-5	10.39 ± 3.81	2.78 ± 0.67	0.99 ± 0.02	29.71 ± 12.56	0.76 ± 0.41	0.85 ± 0.08	2,622.67 ± 42.44	2,652.37 ± 48.00
	Negative control	14.35 ± 1.76	2.89 ± 0.12	1.00 ± 0.00	16.67 ± 12.51	0.95 ± 0.13	0.80 ± 0.03	2,703.33 ± 25.17	2,720.00 ± 27.40

Figure 6E exhibits the equivalent circuit fitting the experimental impedance data. The *Q* represents the non-ideal behavior of the capacitor, and there is a positive correlation between *Q* and capacitance (*C*). The capacity was related to the property of the passive film’s metal/solution interface based on the following equation: *C* = εε_0_A/d, where *C* denotes the capacitance of a film, ε_0_ (8.854 × 10^−14^ F·cm^−1^) is the vacuum permittivity, ε is the dielectric constant, and A and d are the area and thickness of the film, respectively. According to Table 1, *Q*_ou_ is one order of magnitude greater than *Q*_in_, and *R*_ou_ is two orders of magnitude less than *R*_in_. The ε of the outer layer was estimated to be greater than that of the inner layer, indicating that the inner layer is more compact and protective. The corrosion resistance of the passive film (*R*_T_) for each group is manifested in Figure 6F. The *R*_T_ values of titanium treated with peptides (in 48 h) were higher than those of the positive control group (*p* < 0.05), demonstrating that 1018 and DJK-5 could prevent *S. mutans* from deteriorating the passive film. Furthermore, the RT values of titanium treated with 10 μg/mL of DJK-5 were close to the negative control group, denoting a significant anticorrosion efficacy. In the case of two 48 h treatments, the *R*_T_ of titanium treated with peptides increased slightly. This trend was consistent with the results of PDP experiments.

## 4 Discussion

In this work, the effects of peptides 1018 and DJK-5 on the biofilm killing and corrosion behaviors of titanium exposed to *S. mutans* were evaluated. The results demonstrated that peptides 1018 and DJK-5 (at 5 and 10 μg/mL) killed the majority of the bacteria (from 53.95% to 84.82%) in one and two 48-h treatments. Ten μg/mL of DJK-5 inhibited *S. mutans* growth the most and kept the pH neutral. Furthermore, the two peptides exhibited less negative *E*_corr,_ significantly lower *I*_corr_, and higher *R*_T_ than the positive control after one and two 48-h treatments, with 10 μg/mL of DJK-5 showing the greatest effects. These findings suggest that antibiofilm peptides were effective in improving the corrosion resistance of titanium with biofilm-killing ability. Hence, the null hypotheses have to be rejected.

As an opportunistic bacterium known to facilitate the corrosion of dental implants, *S. mutans* has been extensively selected *in vitro* and *in vivo* studies to evaluate the biofilm formation and electrochemical behaviors of titanium (Souza et al., 2018; Chai et al., 2021; Lu et al., 2022). The growth of *S. mutans* in the positive control group remained stable after 48 h, indicating a mature biofilm characteristic (Costa et al., 2020) (which was used as the observation time point in subsequent experiments), and the bacteria were largely alive after one or two 48-h periods of incubation. As the biofilm develops and the pH rises, the accumulation of microbial metabolic byproducts, including lactic acid, and the acidic environment are expected to trigger titanium corrosion. The colonization of oral microbes in both the early and late stages of SLA titanium surface corrosion has been reported in previous investigations (Rodrigues et al., 2016; Siddiqui et al., 2019). Severe corrosive characteristics, such as discoloration and pitting, were discovered after analyzing these retrieved titanium samples. Apart from acid production, recent studies have suggested that the metabolism of viable microbial cells and oxygen consumption contribute to titanium corrosion as well (Zhang et al., 2013; Fukushima et al., 2014). The non-uniform distribution of oxygen contents generated by reactions between oxygen around microbes and their metabolites most likely creates a local potential difference, and thus electrochemically corrosive properties may appear.

Treatment with peptides 1018 and DJK-5 (at 5 and 10 μg/mL) inhibited *S. mutans* growth and killed the biofilms on the titanium surface to varying degrees, and DJK-5 (10 μg/mL) displayed the highest ability among all groups to maintain a neutral pH, prevent microbial growth, and induce cell death within biofilms. These results confirm that bacterial growth inhibition and biofilm killing (with pH stabilization) on titanium can be obtained by antimicrobial peptides, implying an effective strategy for preventing biofilm-induced implant infection and corrosion. The mechanism behind this could be elucidated as follows. In general, the integrity or functioning of bacterial cell membranes can be destroyed by positively charged antimicrobial peptides because of the presence of negatively charged bacterial lipids (Bechinger and Gorr, 2017). Both peptides have been shown to be effective against a variety of Gram-negative and Gram-positive bacteria in terms of killing and decomposing preformed biofilms (Pletzer et al., 2016; Pletzer and Hancock, 2016). In addition to the promotion of ppGpp degradation involved in biofilm development, biofilm killing may be enhanced through immune cell activation, autophagy, and apoptosis mediation (Hancock et al., 2016). Interestingly, peptide DJK-5 outperformed peptide 1018 in terms of antibiofilm capability. In this regard, DJK-5 possesses improved *in vitro* biological activities and is less likely to be recognized by host or bacterial proteases during infections, as well as being more resistant to degradation by host proteases (de la Fuente-Núñez et al., 2016). Furthermore, following an extra 48 h of bacterial growth in a medium with 10 μg/mL of DJK-5, the ratio of dead bacteria to total bacteria dropped in comparison to the initial 48 h. This may be due to the fact that the growth of live bacteria might outpace the prolonged bactericidal effect of DJK-5. However, it is encouraging that DJK-5 was able to kill nearly 80% of the bacterial biofilms after 96 h. Even so, future studies are demanded to explore the long-term biofilm killing efficacy of these peptides.

SEM was used to observe the surface morphology of corrosive titanium disks exposed to *S. mutans* and peptides. Irregular micro-pits of varying were manifested along the grooves in the positive control group (contained *S. mutans* without peptides) after 48 + 48 h, which was consistent with the findings of Souza et al. (2018) and Siddiqui et al. (2019). On the contrary, micro-pits decreased on surfaces treated with peptides 1018 and DJK-5, indicating that the addition of peptides had the potential to prevent titanium corrosion. The inhibitory effects with applied potentials was further assessed by PDP curves. Generally, the electrochemical corrosion of titanium alloys can arise from several modes of attack, each with its unique mechanisms (Nyby et al., 2021). Therefore, multiple parameters are available for assessing the salient features of corrosion events, such as *E*_corr_, *I*_corr_, *E*_pit_, and *I*_passive_. While the passive film slows down the rate of uniform corrosion, it can also accelerate localized corrosion, which is linked to the local breakdown of passive films. The persistence of titanium-based implant may fail as a result of these corrosion mechanisms. The electrochemical data in the positive control group showed the lowest *E*_corr_ and the highest *I*_corr_ and *I*_passive_ among all groups, denoting that the electrochemical properties of titanium can be changed by the grown biofilms. This could be ascribed to the effect of *S. mutans* growth and metabolism at high sucrose concentrations on acid production, which lowers the pH value of the culture medium (Souza et al., 2013; Costa et al., 2021). As a result, the stability of the passive film on titanium deteriorated, and the corrosion and degradation rate of the film in saliva was accelerated. Notably, peptides 1018 and DJK-5 shifted the *E*_corr_ and *E*_pit_ in a more positive direction and displayed significantly lower *I*_corr_ and *I*_passive_ than the positive control (in 48 and 48 + 48 h), with 10 μg/mL of DJK-5 achieving the greatest effects. Thus, the use of peptides effectively reduced the corrosion susceptibility of titanium in the presence of biofilms, which supports the results of biofilm-killing experiments.

The resistance properties of peptides applied to titanium exposed to *S. mutans* were assessed by EIS measurements. The resistance (*R*_T_) of titanium treated with peptides 1018 and DJK-5 (at 5 and 10 μg/mL) in *S. mutans* medium for 48 and 48 + 48 h was significantly increased, and the anticorrosion trend corresponded to the antibiofilm trend. From a clinical standpoint, titanium corrosion and passive film dissolution induced by biofilm challenge are considered the primary causes of titanium ion release (Noronha Oliveira et al., 2018). Such ions, along with debris and particles released by titanium-based dental implants, are harmful to the surrounding oral tissues, triggering inflammatory responses and bone loss (Messous et al., 2021; Barrak et al., 2022). In the present study, the application of peptides was shown to increase the electrochemical stability of titanium. When combined with the results from the PDP, EIS, and antibiofilm experiments, the improved corrosion resistance might relate to their effectiveness in *S. mutans* growth inhibition and biofilm killing. In this regard, the pH of the local environment was kept neutral, and the decrease in the generation of acids and the formation of differential oxygen slowed the corrosive processes. With the reduction of metal dissolution and surface deterioration, the passive film of titanium can be protected to maintain the stability of the implants (Hancock et al., 2021).

The use of antibiofilm peptides was successful in enhancing the corrosion resistance of titanium surfaces against microbial biofilms. The effectiveness of peptides 1018 and DJK-5 on biofilm killing on titanium surfaces at an initial stage was confirmed to reflect clinical situations of early implant infection and treatment. In terms of form, peptides could be applied as functional components to be incorporated into mouthwash or irrigants, preventing and/or treating biofilm-associated implant infection and corrosion to reduce the risk of failure. Despite the encouraging results, it should be noted that there is a complex relationship between microorganisms, corrosion, osseointegration, and surrounding tissues of titanium-based implants. Moreover, it could be a slow process for the deterioration of the passive film caused by microbial accumulation and acid production, and a model system that can mimic more realistic *in vivo* situations should be developed. Thus, further studies, including clinical trials, are needed to explore the detailed mechanism of antibiofilm peptides interacting with titanium and microbial biofilm, as well as their long-term efficacy in overcoming dental implant corrosion.

## 5 Conclusion

This study evaluated the effects of antibiofilm peptides on the corrosion resistance of titanium against *S. mutans* biofilms. The results suggested that the application of antibiofilm peptides effectively killed *S. mutans* biofilms and reduced the corrosion reaction on titanium. Furthermore, 10 μg/mL of DJK-5 can achieve the greatest effects and lead to a pH neutral environment. Therefore, antibiofilm peptides offer promising benefits for promoting the anticorrosion performance of titanium against microbial biofilm to enhance the stability of dental implants and prevent peri-implant infections.

## Data Availability

The original contributions presented in the study are included in the article/Supplementary Material, further inquiries can be directed to the corresponding authors.
