# Supplementary material for: Antibiofilm peptides enhance the corrosion resistance of titanium in the presence of Streptococcus mutans
Source: Front Bioeng Biotechnol. 2024 Jan 10;11:1339912. doi: 10.3389/fbioe.2023.1339912 (PMC10809395; doi:10.3389/fbioe.2023.1339912)
Supplement: Supplementary file 1 [file DataSheet1.PDF]

Supplementary Material

Antibiofilm peptides enhance the corrosion resistance of titanium in the presence of *Streptococcus mutans*

Dan Wang[1,2,†], Yingying Yue[5,†], He Liu[2], Tian Zhang[4], Evan F. Haney[6], Robert E.W. Hancock[6], Jian Yu[2,3*], Ya Shen[2*]

[1]Department of Stomatology, Tongji Hospital, Tongji Medical College, Huazhong University of Science and Technology, Wuhan, China

[2]Division of Endodontics, Department of Oral Biological and Medical Sciences, Faculty of Dentistry, University of British Columbia, Vancouver, Canada

[3]State Key Laboratory of Oral & Maxillofacial Reconstruction and Regeneration, Key Laboratory of Oral Biomedicine Ministry of Education, Hubei Key Laboratory of Stomatology, School & Hospital of Stomatology, Wuhan University, Wuhan, China

[4]School of Medicine, Vanderbilt University, Nashville, United States

[5]Liaoning Institute of Science and Technology, Benxi, China

[6]Centre for Microbial Diseases and Immunity Research, Department of Microbiology and Immunology, University of British Columbia, Vancouver, Canada

*** Correspondence:**
Ya Shen; Jian Yu
yashen@dentistry.ubc.ca; yujiandoctor@whu.edu.cn

[†]These authors contributed equally to this work and designated as co-first authors.

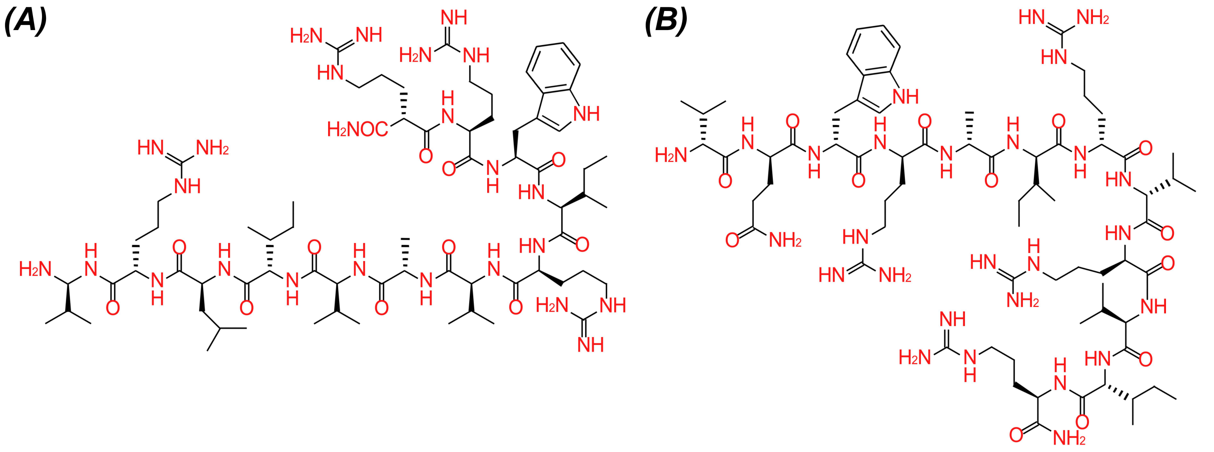

Supplementary Figure S1. Chemical structure of peptides (A)1018 and (B) DJK-5.
